# Case report: Genomic analysis of a therapy-related chronic myelomonocytic leukemia with *KMT2A* rearrangement that progressed to acute myeloid leukemia with acute promyelocytic leukemia-like features

**DOI:** 10.3389/fonc.2023.1116418

**Published:** 2023-02-17

**Authors:** Tomotaka Suzuki, Rui Yokomori, Takaomi Sanda, Takaki Kikuchi, Yoshiaki Marumo, Shiori Kinoshita, Tomoko Narita, Ayako Masaki, Asahi Ito, Masaki Ri, Shigeru Kusumoto, Hirokazu Komatsu, Hiroshi Inagaki, Shinsuke Iida

**Affiliations:** ^1^ Department of Hematology and Oncology, Nagoya City University Graduate School of Medical Sciences, Nagoya, Japan; ^2^ Cancer Science Institute of Singapore, National University of Singapore, Singapore, Singapore; ^3^ Department of Pathology and Molecular Diagnosis, Nagoya City University Graduate School of Medical Sciences, Nagoya, Japan

**Keywords:** case report, chronic myelomonocytic leukemia, therapy-related myeloid neoplasm, *KMT2A*, *NRAS*

## Abstract

We report a 69-year-old female who was a human T-cell leukemia virus type 1 carrier and exhibited a unique clinical course of developing three hematological malignancies within a short period: diffuse large B-cell lymphoma (DLBCL), chronic myelomonocytic leukemia (CMMoL), and acute myeloid leukemia (AML). Although the blast cells in AML showed typical morphological and immunophenotypical features of acute promyelocytic leukemia (APL), it did not harbor *RARα* gene fusion and thus initially diagnosed as APL-like leukemia (APLL). The patient developed heart failure with a fulminant clinical course and died soon after the diagnosis of APLL. Retrospective analysis with whole-genome sequencing detected a chromosomal rearrangement between *KMT2A* and *ACTN4* gene loci both in CMMoL and APLL samples, but not in the DLBCL sample. Therefore, CMMoL and APLL were considered to be derived from the same clone with *KMT2A* translocation associated with prior immunochemotherapy. However, *KMT2A* rearrangement is rarely found in CMMoL in general and *ACTN4* is also a rare partner of *KMT2A* translocation. Thus, this case did not follow typical transformational process of CMMoL or *KMT2A*-rearranged leukemia. Importantly, additional genetic alterations, including *NRAS G12* mutation, were found in APLL, but not in CMMoL samples, suggesting that they might contribute to leukemic transformation. This report highlights the diverse effects of *KMT2A* translocation and *NRAS* mutation on the transformation of hematological cells as well as the importance of upfront sequencing analysis to detect genetic backgrounds for a better understanding of therapy-related leukemia.

## 1 Introduction

The development of secondary malignancies is a major clinical problem for cancer survivors who receive intensive chemotherapy for primary malignancies. Treatment with cytotoxic agents, such as topoisomerase II inhibitors (e.g., etoposide and doxorubicin), is known to be the causative factor of therapy-related acute myeloid leukemia (AML), which typically occurs within 1–3 years after the exposure of topoisomerase II inhibitors without passing through myelodysplastic syndrome or myeloproliferative neoplasms. Other myeloid neoplasms, including chronic myelomonocytic leukemia (CMMoL), have also been reported to be therapy-related malignancies.

Herein, we report a female patient who sequentially developed three different types of hematological malignancies (diffuse large B-cell lymphoma [DLBCL], CMMoL, and acute promyelocytic-like leukemia [APLL]) within 3 years. This patient was a human T-cell leukemia virus type 1 (HTLV-1) carrier but did not develop HTLV-1-related disorders. Retrospective analysis with whole-genome sequencing (WGS) of a series of tumor samples detected a chromosomal rearrangement involving the *KMT2A* gene locus that translocated to *ACTN4* gene locus, a rare partner of translocation, which was detected in CMMoL and APLL samples. Although *KMT2A*-rearrangement has been well known to be associated with therapy-related leukemia, it is rarely found in CMMoL (1). Additionally, although the blast cells in the third malignancy showed all typical morphological and immunophenotypical features of APL, it did not harbor *RARα* gene fusion. Thus, CMMoL or APLL did not possess typical genetic abnormalities that can confirm the diagnosis and instead followed atypical transformation process likely induced by *KMT2A-ACTN4* translocation.

## 2 Case description

A 69-year-old Japanese woman developed neck lymph node enlargement in November 2017. She was a HTLV-1 carrier but was otherwise healthy previously, without family histories of any hematological malignancies. See the supplemental information for more detailed clinical course and supporting findings (**Figures S1**-**S8**). Based on pathological findings of the neck tumor, she was diagnosed with DLBCL of high risk in international prognostic index, and rituximab, cyclophosphamide, doxorubicin, and prednisone (R-CHOP) therapy was administered. She ended R-CHOP therapy for up to 7 cycles owing to adverse events, including several episodes of febrile neutropenia and anemia requiring blood transfusion. She achieved a partial metabolic response based on positron emission tomography findings of residual fluorodeoxyglucose accumulation in the neck region at the end of R-CHOP therapy; the disease did not relapse thereafter.

Six months after the last R-CHOP cycle, a second hematological malignancy developed. The patient presented with a high fever that was refractory to antibiotics and lasted for 2 weeks. A peripheral blood sample showed remarkable monocytosis. Bone marrow (BM) samples showed a marked increase in monocytes, ranging from immature to mature stages. She was diagnosed with CMMoL (**Table S1**), and azacitidine was administered. Monocytosis gradually resolved after three cycles of azacytidine; however, the patient developed cholecystitis and discontinued azacitidine, although cholecystitis was not considered to be related to azacitidine therapy.

**Figure 1:**
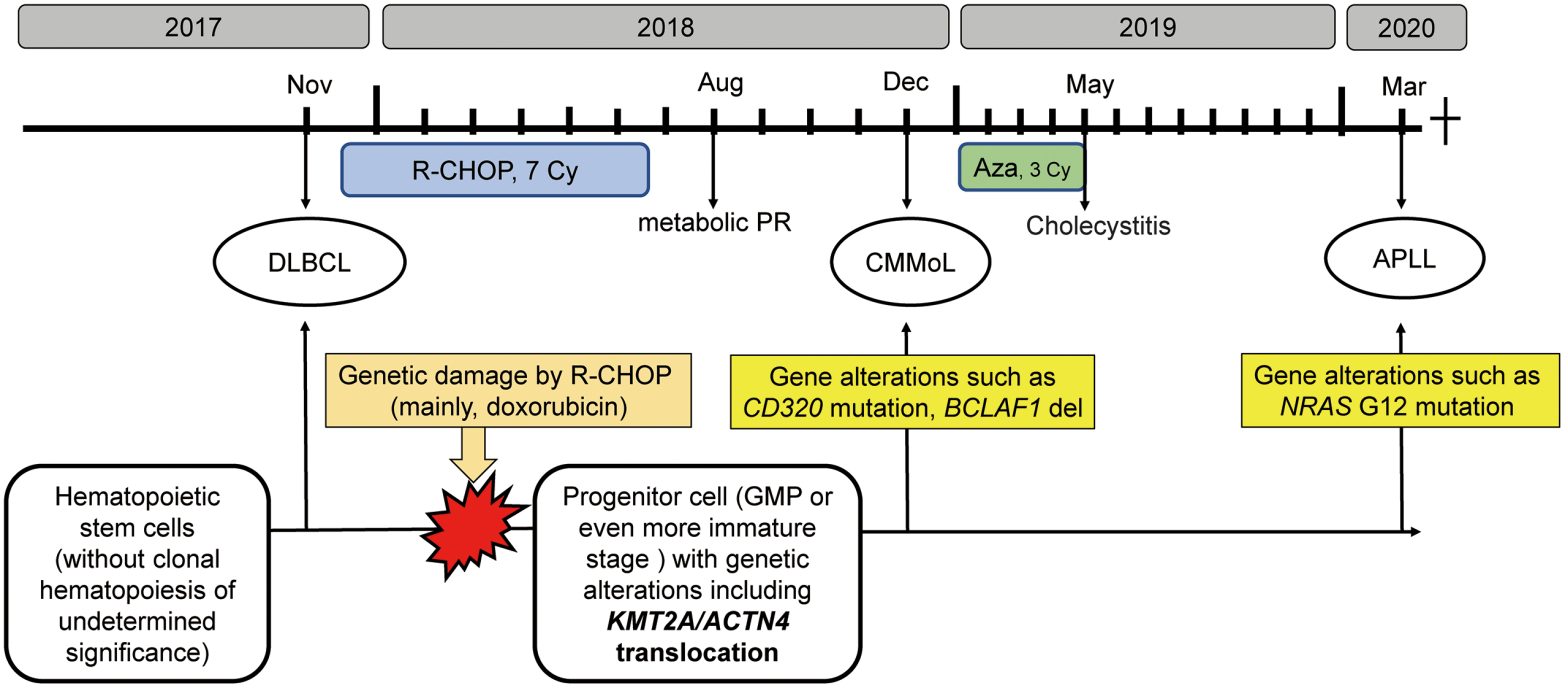
The clinical course along with schematic diagram of possible genetic backgrounds of the series of hematological malignancies The R-CHOP therapy might introduce genetic alterations, such as *KMT2A* translocation in progenitor cells, from which CMMoL and APLL were derived. In addition, acquiring genetic mutation in *NRAS* resulted in leukemic translation. R-CHOP, rituximab, cyclophosphamide, doxorubicin, vincristine, and prednisolone; Aza, azacitidine; DLBCL, diffuse large B-cell lymphoma; CMMoL, chronic myelomonocytic leukemia; APLL, acute promyelocytic-like leukemia; PR, partial response; GMP, granulocyte/monocyte progenitors.

Eleven months after the last treatment with azacitidine, a third hematological malignancy developed. The patient presented with pancytopenia. The BM sample showed a marked increase in the number of promyelocytes. Flow cytometry revealed that these cells were positive for CD13, CD33, and MPO and negative for CD34 and HLA-DR, which is a typical immunophenotypical pattern of acute promyelocytic leukemia (APL). Thus, all-*trans* retinoic acid was administered. However, G-banding analysis of the BM sample showed a normal karyotype, and fluorescence *in situ* hybridization (FISH) analysis did not show *PML-RARA* fusion. Hence, we have diagnosed as APLL. However, she developed heart failure with a fulminant clinical course, which might have been caused by Takotsubo cardiomyopathy or myocardial infarction secondary to disseminated intravascular coagulation, and she died soon after the diagnosis. Overview of the clinical course of this patient, along with the possible genetic backgrounds of the series of hematological malignancies, is presented in **Figure 1**.

## 3 Diagnostic assessment

Because CMMoL and APLL did not exhibit typical cytogenetic abnormalities, we retrospectively investigated the mutational profiles by WGS analysis using genomic DNAs (gDNAs) from each hematological disease (DLBCL, CMMoL, and APLL) and a somatic control (buccal swab). We detected single nucleotide variants (SNVs) and structural variants (SVs) that are present specifically in tumor samples. We also analyzed SNVs that were found in the buccal swab, but not in the reference human genome, to detect potential germline mutations. See the supplemental methods for detailed information on sample preparation, WGS, and bioinformatic analyses.

**Figure 2:**
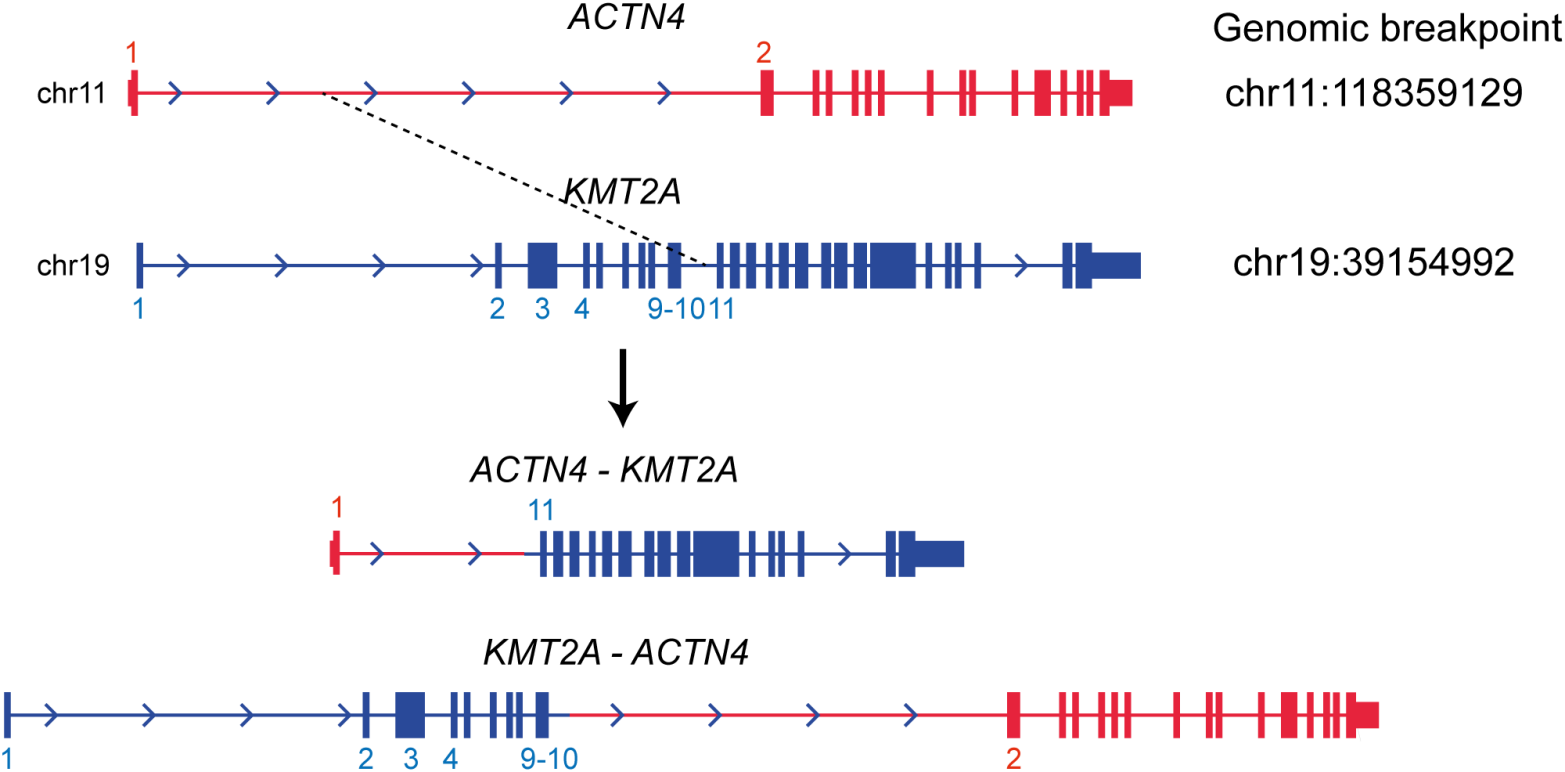
Translocation between KMT2A and ACTN4 genes. It was commonly detected in the CMMoL and APLL samples.

WGS analysis of the buccal swab gDNA revealed no notable germline SNVs known to be cancer predisposing factors. Integration of the HTLV-1 genome was not observed in any of the tumor gDNAs. Thus, DLBCL was considered a sporadic case. Importantly, neither SNVs nor SVs were shared between the DLBCL and CMMoL samples (**Tables S2**, **S3**), and most of the genetic mutations detected in the CMMoL sample were also found in the APLL sample (**Tables 1**, **S2**). No typical genetic mutations associated with clonal hematopoiesis of insignificant potential (CHIP) was identified in any of the samples. This result indicated that CMMoL and APLL cells were derived from the same clone. Notably, WGS analysis was able to detect a chromosomal translocation involving 11q23.3 (*KMT2A*) and 19q13.2 (*ACTN4*) loci, both in CMMoL and APLL samples (**Figure 2** and **Table S3**); this is the only SV that was detected commonly in CMMoL and APLL samples. Independent validation by spectral karyotyping (SKY) using Carnoy fluid from CMMoL and APLL samples also supported this finding; translocation of chromosomes 11 and 19 was detected (**Figures S5**, **S8**). Hence, according to the fifth edition of the WHO Classification of Haematolymphoid Tumours (2), APLL can be re-diagnosed as “AML with *KMT2A* rearrangement”.

**Table 1:** Single nucleotide variants detected in CMMoL and/or APL-like leukemia samples.

	Gene	RefSeq ID, HGVS.p	VAF		Position
			APLL	CMMoL	
Shared mutation between CMMoL and APLL	*ADCY6*	NM_015270.5, A310S	0.417	0.548	chr12:49171977
	*ESPL1*	NM_012291.5, R699W	0.304	0.407	chr12:53671263
	*TRHDE*	NM_013381.2, A356E	0.411	0.469	chr12:72771788
	*MAGEL2*	NM_019066.5, R445H	0.476	0.579	chr15:23891556
	*GPR179*	NM_001004334.4, G1088E	0.724	0.433	chr17:36486189
	*WDR7*	NM_015285.2/NM_052834.2. R884L	0.428	0.433	chr18:54424475
	*MAST3*	NM_015016.2, V568I	0.557	0.467	chr19:18245711
	*MAP7D1*	NM_001286366.2/NM_018067.5, V253M	0.572	0.484	chr1:36640516
	*JAG1*	NM_000214.3, S746G	0.308	0.413	chr20:10625619
	*NINL*	NM_001318226.1/NM_025176.6, V303M	0.615	0.45	chr20:25481601
	*CACNA2D3*	NM_018398.3, L841M	0.429	0.493	chr3:55003836
	*PCDH18*	NM_001300828.2/NM_019035.5, E786Q	0.35	0.444	chr4:138450887
	*PCDHGB3*	NM_018924.5/NM_032097.3, N165S	0.467	0.502	chr5:140750455
	*BTN1A1*	NM_001732.3, P496S	0.311	0.357	chr6:26509307
	*CNOT4*	NM_001008225.2/NM_001190849.2, K394E (A)	0.333	0.622	chr7:135079108
	*CYP2C18*	NM_000772.3/NM_001128925.2, N107T	0.307	0.469	chr10:96447678
	*SH2D3C*	NM_170600.3, H169Q	0.285	0.48	chr9:130536277
	*PIGO*	NM_001201484.1/NM_152850.3, L645F (B)	0.367	0.461	chr9:35089175
	*KMT2A*	NM_001197104.2/NM_005933.4, P1430R	0.267	0.488	chr11:118355647
	*DNAJC22*	NM_001304944.2/NM_024902.4, Y24*	0.334	0.473	chr12:49742727
CMMoL only	*CD320*	NM_016579.4, V68M	ND	0.422	chr19:8369981
APL-like leukemia only	*NRAS*	NM_002524.5, G12D	0.437	ND	chr1:115258747
	*ABCA6*	NM_080284.3, M255I	0.305	ND	chr17:67129808
	*HMCN1*	NM_031935.3, L562V	0.3	ND	chr1:185902812
	*FRMPD4*	NM_001368395.2/NM_001368398.2, D737H (C)	0.437	ND	chrX:12734676
	*MAP3K15*	NM_001001671.3, G664A	0.333	ND	chrX:19416419

HGVS, Human Genome Variation Society; CMMoL, chronic myelomonocytic leukemia; APLL, acute promyelocytic-like leukemia; VAF, variant allele fraction; ND, not detected.

Additional information: (A) NM_013316.4, K394E, NM_001190847.2/NM_001190848.1/NM_001190850.1, K397E. (B) NM_032634.4, L1062F. (C) NM_001368396.2, D702H; NM_001368397.1/NM_014728.3, D700H; NM_001368399.2, Asp697His; NM_001368400.2, Asp660His; NM_001368401.1/NM_001368402.2, D692H. *, stop codon.

We further selected genetic alterations which were specifically found in either CMMoL or APLL sample. Interestingly, an SNV in the *CD320* gene was detected only in the CMMoL sample, whereas several SNVs were detected in only the APLL sample, including *NRAS, ABCA6, HMCN1, FRMPD4*, and *MAP3K15* (**Tables 1**, **S2**). The latter abnormalities potentially contributed to the transformation of APLL cells.

## 4 Discussion

The patient demonstrated a unique clinical course: a series of hematological malignancies (DLBCL, CMMoL, and APLL) occurred within a short period of time. Hence, we hypothesized that these diseases might be related to each other or that the patient might harbor predisposing cancer risks. However, no notable germline mutations or HTLV-1 integration were observed, although HTLV-1 infection might potentially affect anti-tumor immunity. In contrast, our WGS analysis detected *KMT2A-ACTN4* translocation, and several other genetic alterations commonly found in CMMoL and APLL samples but not in DLBCL samples. These results suggest that CMMoL and APLL were derived from a common clone that has somatically acquired *KMT2A-ACTN4* translocation after immunochemotherapy for DLBCL. APLL clones may have evolved by acquiring additional genetic abnormalities including *NRAS G12* mutation.

*KMT2A* translocation has been reported in patients with therapy-related leukemia, and exposure to topoisomerase-II inhibitors is a risk factor (3). However, *KMT2A* rearrangement was initially not detected using the conventional method (i.e., karyotyping). Also, because *KMT2A* rearrangement is rare in CMMoL (1), a targeted approach (e.g., FISH and SKY) or WGS was not considered upfront. Hence, it was originally difficult to suspect or detect *KMT2A* translocation at the point of diagnosis with CMMoL. Patnaik et al. evaluated genetic and cytogenetic alterations in 492 patients with CMMoL, including 45 therapy-related CMMoLs, using targeted capture sequencing. Although CHIP-associated genes *TET2* and *ASXL1* were the two most frequent mutations, translocation involving *KMT2A* was observed in only one patient (1). It is noteworthy that various combinations of somatic mutations involving epigenetic regulation, spliceosome, and signal transduction genes have been associated with CMMoL (2). *KMT2A* encodes a histone lysine methyltransferase that modifies epigenetic status, which is associated with transcriptional activation (4). Hence, a potential explanation is that a single genetic abnormality, namely, *KMT2A* translocation, might cause dramatic epigenetic rewiring of the gene expression program that led to the formation of CMMoL.

Identification of the fusion partner of *KMT2A*, of which >80 genes are known, is also desirable because it provides information for prognosis and disease monitoring (2). In our case, *ACTN4*, which encodes a ubiquitously expressed actin-bundling protein, was translocated to the *KMT2A* locus. *ACTN4* is a very rare partner of *KMT2A* translocation; only two cases, therapy-related B-acute lymphoblastic leukemia in a 69-year-old woman and therapy-related MDS in a 5-year-old man, have been reported to date (5). Further investigation is needed to clarify the significance of *KMT2A/ACTN4* translocation.

During normal development, monocytes and granulocytes are derived from granulocyte/monocyte progenitors (GMP). Hence, we hypothesized that GMP or even more immature stages that harbor the *KMT2A* translocation might be the origin of CMMoL and APLL clones. Given that the APLL sample had many other genetic abnormalities, additional abnormalities might be needed for the leukemic transformation of CMMoL to APLL. One such candidate is the *NRAS G12* mutation (6). Carr et al. reported that in 48 patients who were initially diagnosed with CMMoL but eventually progressed to AML, whole-exome sequencing analysis of paired CMMoL and AML samples revealed that *NRAS* was the main gene whose mutation rate increased from CMMoL to leukemic transformation (7). Notably, in AML with *KMT2A* rearrangements, blast cells usually demonstrate monocytic differentiation (2). However, the blast cells in our case showed typical morphological and immunophenotypical features of APL but did not harbor the *RARα* fusion. Interestingly, some cases of APLL have possessed *KMT2A* translocation without *RARα* translocation (**Table 2**) (8–11). Therefore, the combination of *KMT2A* translocation and additional genetic mutations, e.g., *NRAS* mutations, may contribute to APLL formation.

**Table 2:** Previous reports and our cases of acute promyelocytic-like leukemia with *KMT2A* translocation.

	Case 1	Case 2	Case 3	Case 4	Case 5	Our case
Age	33	57	11	1	0	70
Sex	Female	Male	Male	Male	Male	Female
Partner gene of *KMT2A* translocation	*ELL*	Unspecified	Unspecified	*MLLT11, RPRD2*	*ELL*	*ACTN4*
G-banding analysis results	46 XX	46XY, t(11;17)(q23;q25)	46XY, t(X;11)(q24;q23)	46XY, t(1;11)(q21;q23)	46XY	46XX
Therapy-related/previous disease	No/NA	No/NA	Yes (DOX, AraC, MTX, VCR, and 6-MP)/APL	No information	No information	Yes (R, CY, DOX, VCR and PSL)/DLBCL
Immunophenotype, positive by FCM	CD11b, CD13, CD33, CD38, CD45, CD64, CD117, HLA-DR (dim), CD123 (partial)	CD13, CD15, CD33, CD117, HLA-DR (dim)	CD4, CD13, CD33	NA	NA	CD4, CD13, CD33, CD38, CD117, MPO
Immunophenotype, negative by FCM	CD14, CD34	CD11b, CD14, CD34, CD56, CD64	CD11b, CD34, CD56, HLA-DR	NA	NA	CD14, CD34, CD56, HLA-DR, TdT
Genetic mutations	Negative for mutation (*FLT3-ITD, NPM1, CEBPA, IDH1/2, DNMT3A and c-kit*)	NA	NA	*DGKH, NRAS*	*SPI1*	*NRAS, ABCA6, HMCN1, FRMPD4*, *MAP3K15*
ATRA use/effect	No information	Not used	Yes/not effective	No information	No information	Yes/not evaluable
Reference	(8)	(9)	(10)	(11)	(11)	

NA, not available/applicable; DOX, doxorubicin; AraC, cytarabine; MTX, methotrexate; VCR, vincristine; 6-MP, mercaptopurine; APL, acute promyelocytic leukemia; R, rituximab; CY, cyclophosphamide; PSL, prednisolone; DLBCL, diffuse large B-cell lymphoma; CMMoL, chronic myelomonocytic leukemia; FCM, flow cytometry; ATRA, all-trans retinoic acid.

In conclusion, this report highlights the diverse effects of *KMT2A* translocation on the transformation of hematological cells and the potential effect of *NRAS* mutations on the evolution of leukemic clones. Upfront WGS analysis may be considered, especially for suspected cases of secondary hematological malignancies, to unbiasedly detect genetic alterations and understand the transformational process.

## 5 Patient perspective

The patient received explanation of her medical condition and agreed to receive treatments. The patient hoped to know the reason of developing multiple hematological malignancies.

## Data availability statement

The datasets presented in this article are not readily available because of ethical/privacy restrictions. Requests to access the datasets should be directed to the corresponding author.

## Ethics statement

This study was approved by the institutional review board of Nagoya City University Hospital. Written informed consent was obtained from the patient to use her samples for genetic analyses. This study was conducted in accordance with the principles of the Declaration of Helsinki. The patient provided their written informed consent to participate in this study. Written informed consent was obtained from the individual for the publication of any potentially identifiable images or data included in this article.

## Author contributions

TSu, RY and TSa designed the study and wrote the manuscript. TSu extracted the genomic DNA. RY performed the bioinformatic analyses. TSu, RY, and TSa interpreted the sequencing data. TK, YM, SKi, TN, AI, MR, SKu, HK, and SI were responsible for patient medical care. AM and HI were responsible for pathological diagnosis. All authors contributed to the article and approved the submitted version.
