# Supplementary material for: Case report: Genomic analysis of a therapy-related chronic myelomonocytic leukemia with KMT2A rearrangement that progressed to acute myeloid leukemia with acute promyelocytic leukemia-like features
Source: Front Oncol. 2023 Feb 17;13:1116418. doi: 10.3389/fonc.2023.1116418 (PMC9981998; doi:10.3389/fonc.2023.1116418)
Supplement: Supplementary file 1 [file DataSheet_1.zip › Suzuki et al Supplemental Materials/Data_Sheet.docx]

Data Sheet

**Case Report: Genomic analysis of a therapy-related chronic myelomonocytic leukemia with *KMT2A* rearrangement that progressed to acute myeloid leukemia with acute promyelocytic leukemia-like features**

**Tomotaka Suzuki^1†^, Rui Yokomori^2†^, Takaomi Sanda^1,2*^, Takaki Kikuchi^1^, Yoshiaki Marumo^1^, Shiori Kinoshita^1^, Tomoko Narita^1^, Ayako Masaki^3^, Asahi Ito^1^, Masaki Ri^1^, Shigeru Kusumoto^1^, Hirokazu Komatsu^1^, Hiroshi Inagaki^3^ and Shinsuke Iida^1^**

**Patient’s clinical course**

## **1. *Diffuse large B-cell lymphoma (DLBCL)***

The patient developed neck lymph node enlargement in November 2017, when she was 69 years of age. She was a human T-cell leukemia virus type 1 (HTLV-1) carrier but did not develop HTLV-1-related disorders. On the basis of the pathological findings of the neck tumor, the patient was diagnosed with DLBCL.

**International prognostic index: high**

- Ann Arbor stage IV (the disease involved systemic lymph nodes, bone marrow, and the stomach)
- Number of extranodal lesions: 2 (bone marrow and the stomach)
- Performance status at diagnosis: 1
- Elevated lactate dehydrogenase level at diagnosis: 345 U/L
- Age: 69 years

**Results of G-band analysis**

- Bone marrow sample: normal karyotype
- Neck lymph node sample: not evaluable because cells in the interphase were unavailable

**Results of flow cytometric analysis** (Figure S1)


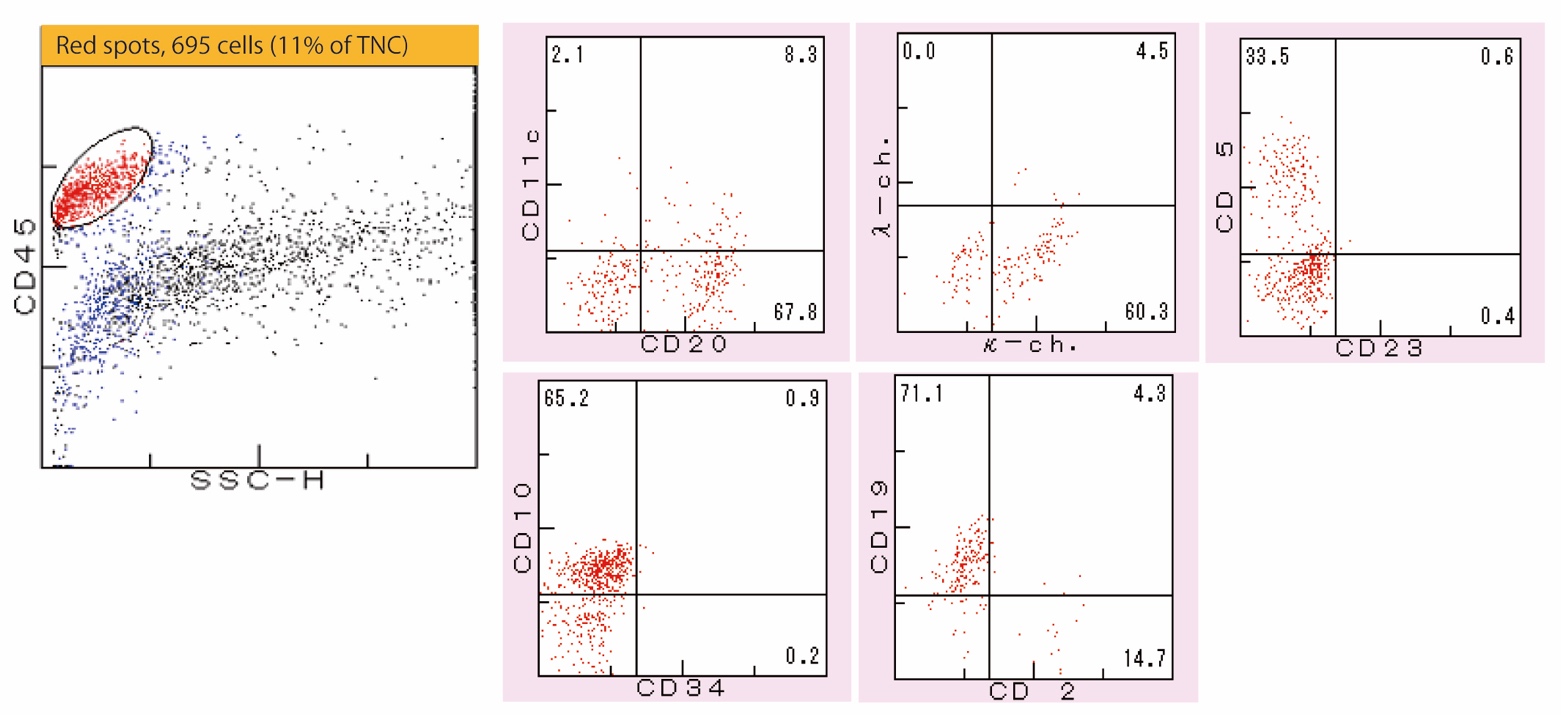


**Figure S1.** Flow cytometric analysis of the involved lymph nodes

Red spots indicate lymphoma cells, which were positive for CD10, CD19, and CD20 and negative for CD5 and CD23.

Abbreviations: TNC, total nucleated cell; SSC, side scatter.


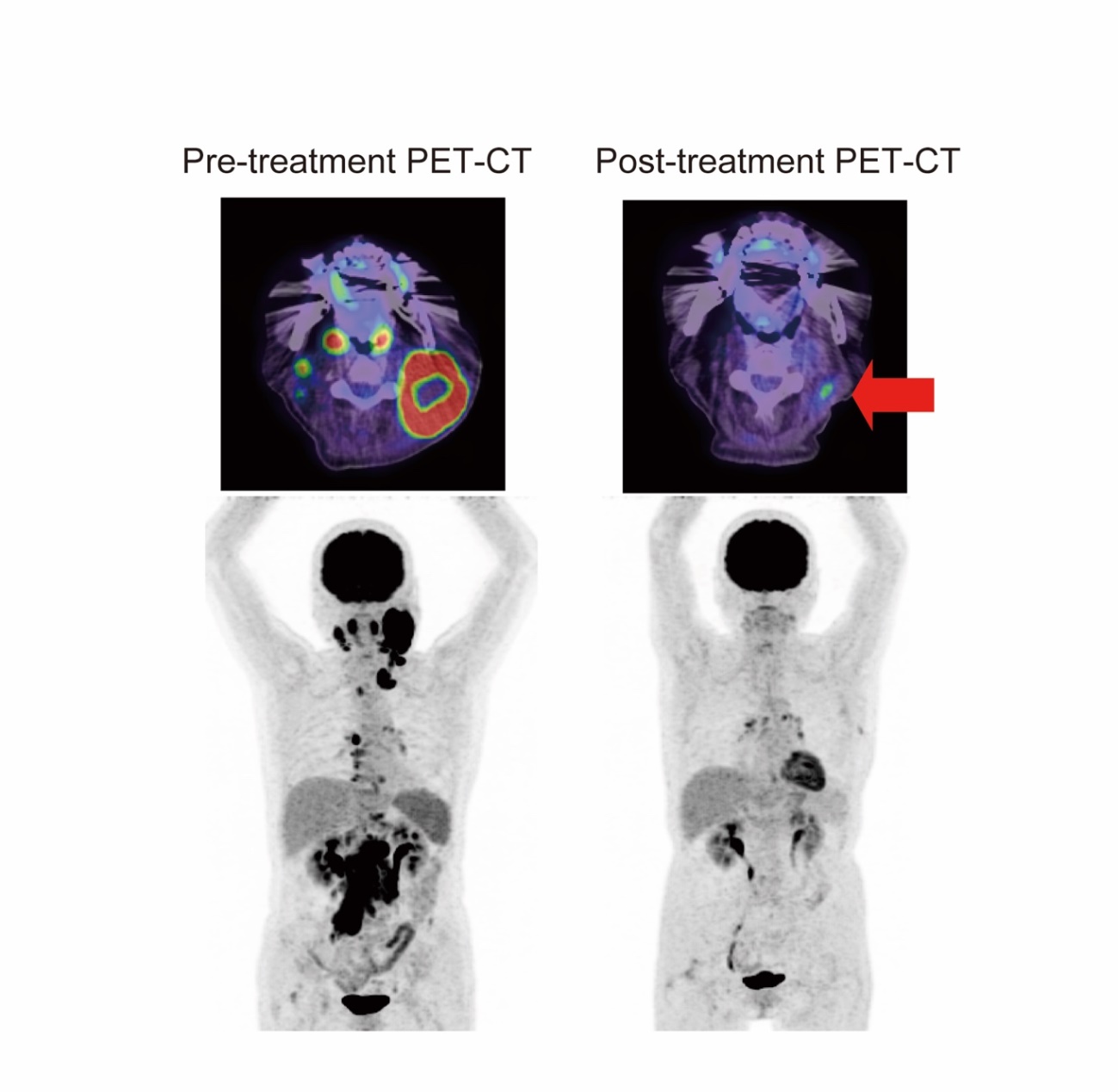


**Figure S2**. Positron emission tomography (PET)-computed tomography (CT) findings

Left side: PET-CT image of pre-treatment. Right side: post-treatment PET-CT image. A slight fluorodeoxyglucose uptake is detected in the neck lymph node.

**2. *Chronic myelomonocytic leukemia (CMMoL)***

Six months after the last R-CHOP cycle for DLBCL, the patient presented with a high fever that was refractory to antibiotics and lasted for 2 weeks. A peripheral blood sample revealed remarkable monocytosis. The bone marrow sample showed a marked increase in monocytes, ranging from the immature to mature stages (Figure S3).

**Complete blood cell counts**

White blood cell, 10.6 × 10^9^/L (blasts, 0%; neutrophils, 2%; monocytes, 94%; lymphocytes, 4%); hemoglobin level, 8.8 g/dL; hematocrit level, 27%; mean corpuscular volume (MCV), 97.5 fl; platelet count, 54 × 10^9^/L.

**Bone marrow smear findings**


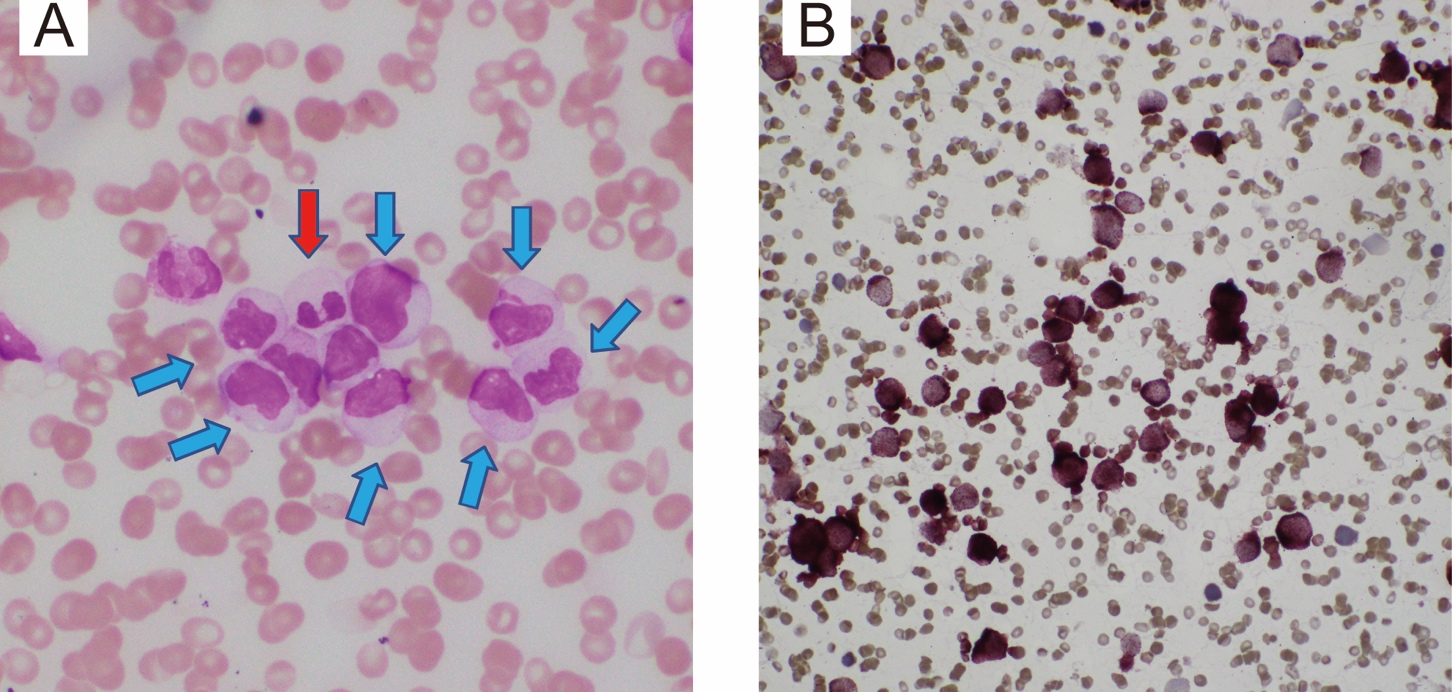


**Figure S3.** Bone marrow smear findings. (A) Red arrow, dysplastic neutrophils with pseudo-Pelger-Huet anomalies; blue arrow, monocytes with immature to mature morphology. (Giemsa stain, original magnitude ×400) B: Almost all infiltrating cells were positive for non-specific esterase staining (original magnitude ×400).

**Results of flow cytometric analysis** (Figure S4)


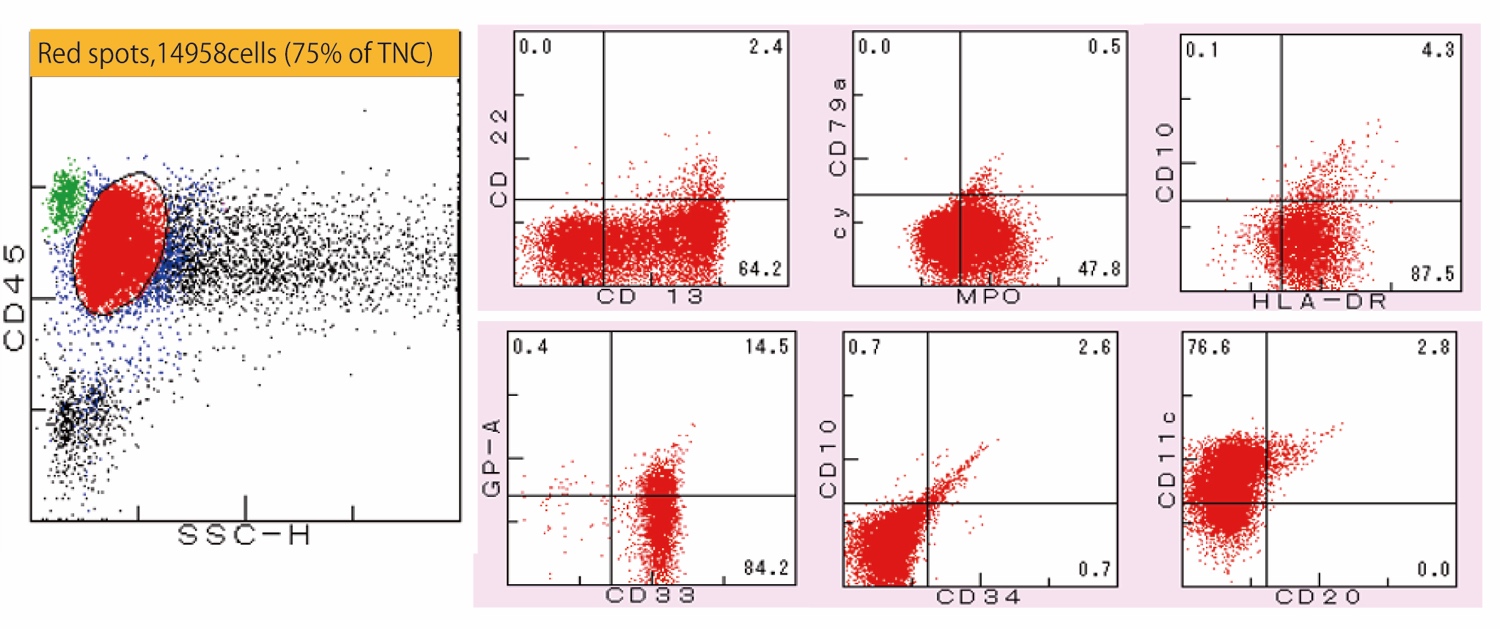


**Figure S4.** Flow cytometric analysis of the bone marrow samples. Red spots indicate cells of the monocyte lineage, which were positive for CD11c, CD13, CD14, MPO, and CD4 (dim).

Abbreviations: TNC, total nucleated cell; SSC, side scatter.

**Results of spectral karyotyping** (Figure S5)


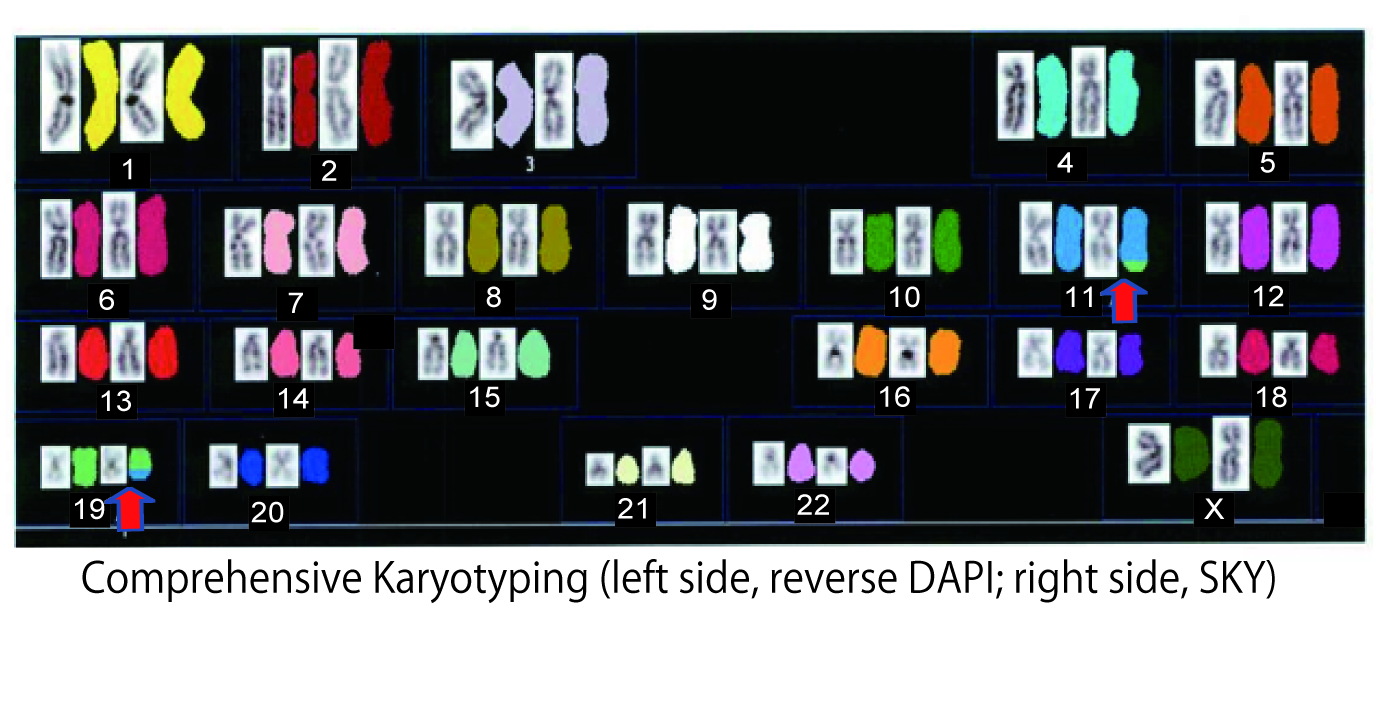


**Figure S5.** Spectral karyotyping of bone marrow samples.

Translocations of chromosomes 11 and 19 are identified (red arrows). The size of chromosomes 11 and 19 did not change after translocation.

**Other laboratory test results**

- G-band analysis of the bone marrow samples with normal karyotypes
- *FIP1L1-PDGFRα* translocation in bone marrow samples is not detected by fluorescence in situ hybridization.
- *The JAK2 V617F* mutation was not detected.
- Chimeric messenger RNA screening test result: No detection of Major-*BCR/ABL,* Minor-*BCR/ABL, PML/RARA, AML1/MTG8, CBFβ/MYH11, DEK/CAN, NUP98/HOXA9, ETV6/AML1, E2A/PBX1, SIL/TAL1, MLL/AF4, MLL/AF6, MLL/AF9,* and *MLL/ENL*

## **3. *Acute promyelocyte-like leukemia (APLL)***

Eleven months after the last azacitidine treatment, the patient presented with pancytopenia. A bone marrow sample showed a marked increase in the number of promyelocytes.

**Complete blood cell count and biochemical test results**

WBC count, 1.0 × 10^9^/L (blasts, 0%; neutrophils, 9%; eosinophils, 1%; monocytes, 8%; lymphocytes, 82%; erythroblasts, 1%); hemoglobin level, 7.2 g/dL; hematocrit level, 20.4%; MCV, 108.8 fl; platelet count, 35 × 10^9^/L; fibrin/fibrinogen degradation product level, 5.6 µg/mL; lactate dehydrogenase level, 310 U/L

**Bone marrow smear findings** (Figure S6)

***
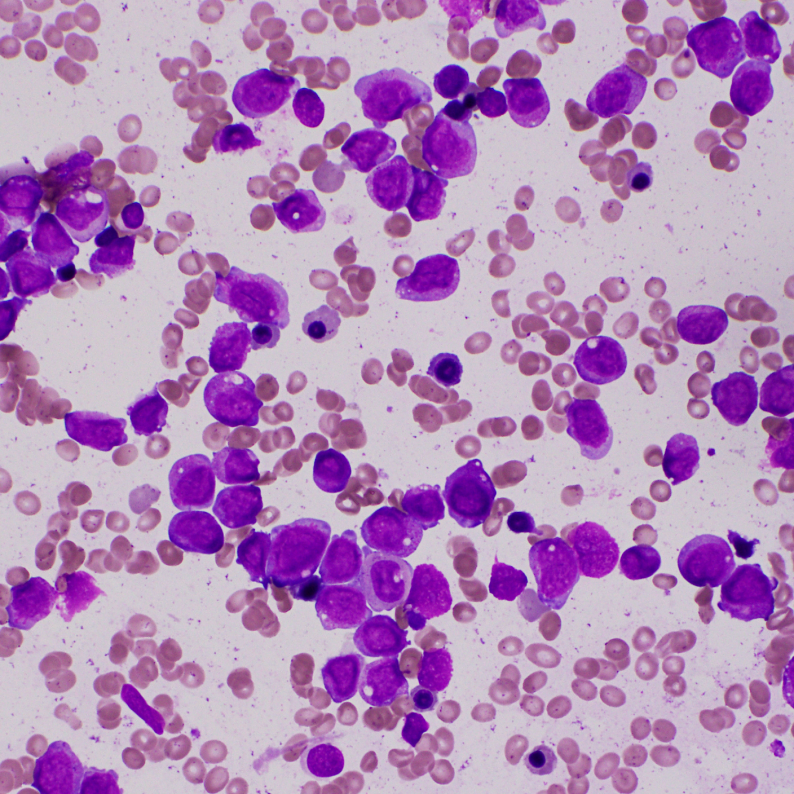
***

**Figure S6**. Bone marrow smear findings. Prominent infiltration of promyelocytes with atypia is observed. Fagot cells are not detected (Giemsa stain, original magnification ×400).

**Results of flow cytometric analysis** (Figure S7)

**
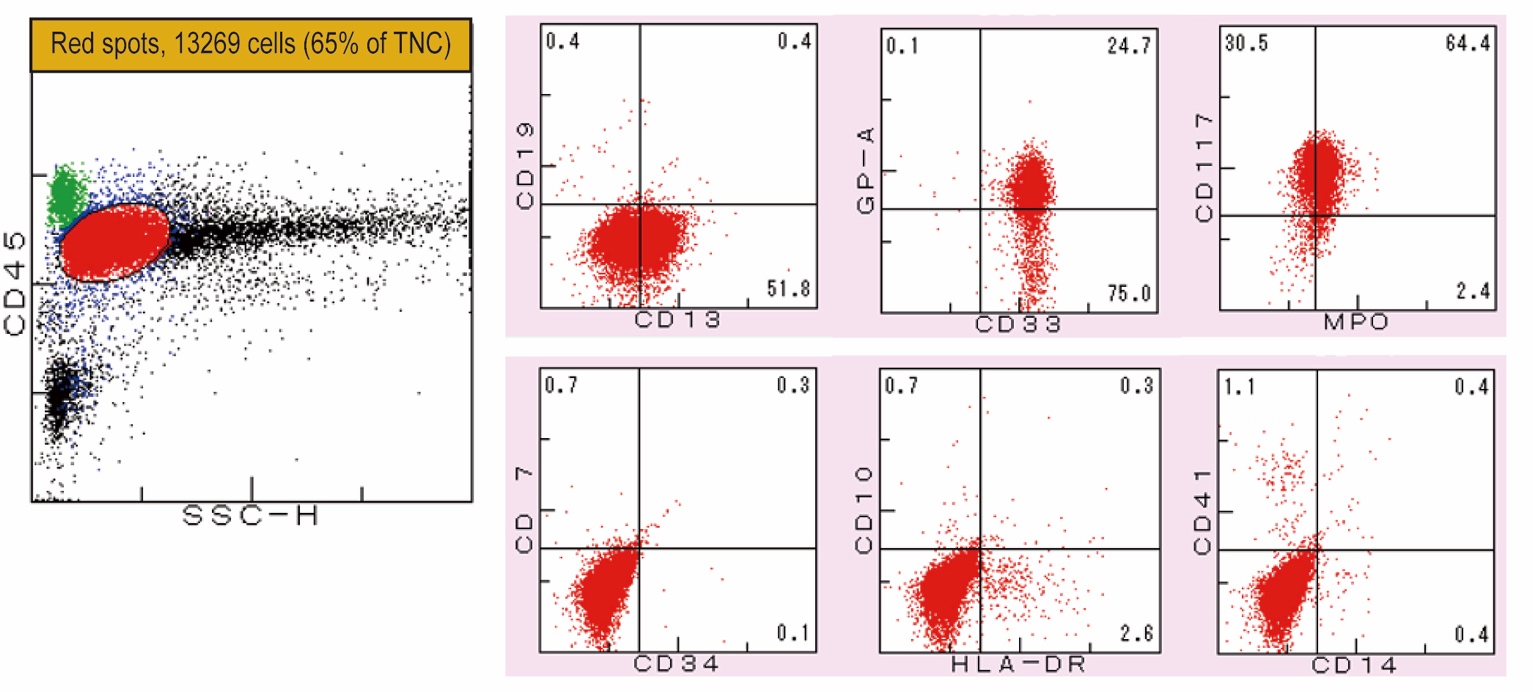
**

**Figure S7**. Flow cytometric analysis of the bone marrow sample. Red spots indicate promyelocytes, which were positive for CD13, CD33, MPO, CD117, and CD4 (dim). The cells were negative for CD14, CD34, and HLA-DR.

Abbreviations: TNC, total nucleated cell; SSC, side scatter.

**Results of spectral karyotyping** (Figure S8)

**
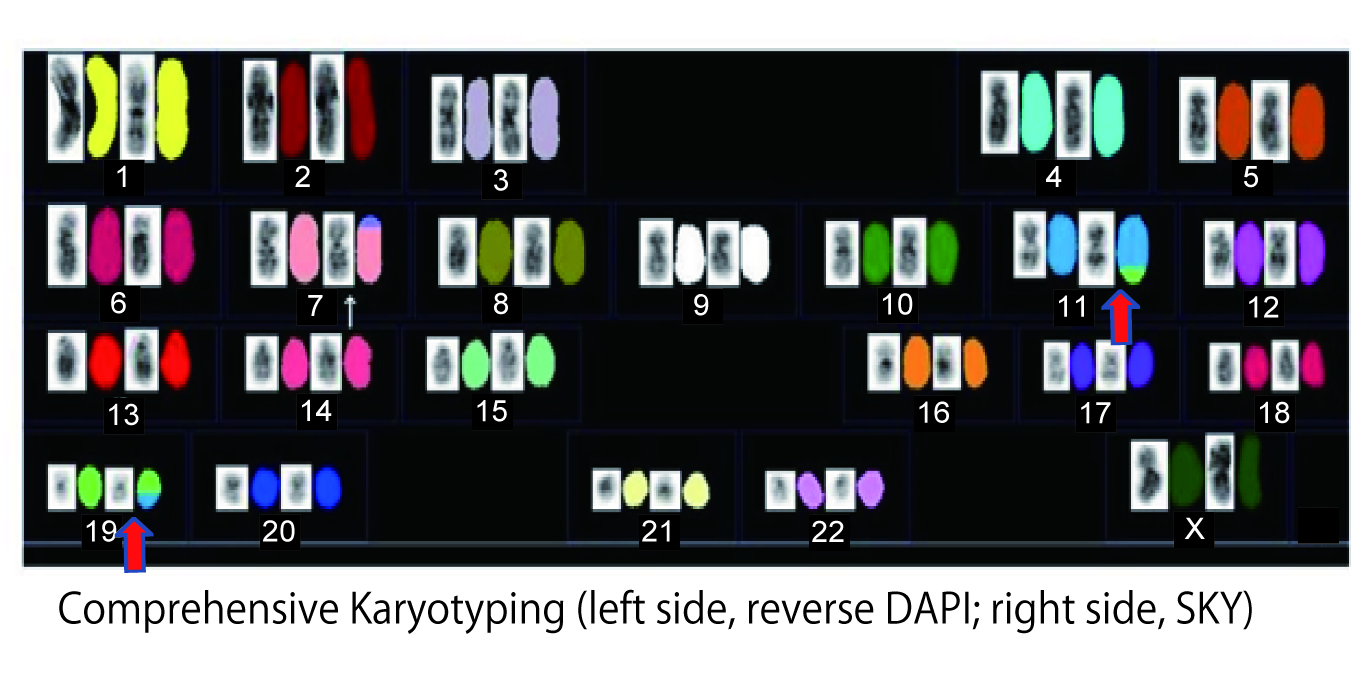
**

**Figure S8**. Spectral karyotyping of the bone marrow sample.

Translocations of chromosomes 11 and 19 are identified (red arrows). The size of chromosomes 11 and 19 did not change after translocation.
