# Supplementary material for: Case report: Genomic analysis of a therapy-related chronic myelomonocytic leukemia with KMT2A rearrangement that progressed to acute myeloid leukemia with acute promyelocytic leukemia-like features
Source: Front Oncol. 2023 Feb 17;13:1116418. doi: 10.3389/fonc.2023.1116418 (PMC9981998; doi:10.3389/fonc.2023.1116418)
Supplement: Supplementary file 1 [file DataSheet_1.zip › Suzuki et al Supplemental Materials/Supplementary_Methods.docx]

Supplementary Methods

**Case Report: Genomic analysis of a therapy-related chronic myelomonocytic leukemia with *KMT2A* rearrangement that progressed to acute myeloid leukemia with acute promyelocytic leukemia-like features**

**Tomotaka Suzuki^1†^, Rui Yokomori^2†^, Takaomi Sanda^1,2*^, Takaki Kikuchi^1^, Yoshiaki Marumo^1^, Shiori Kinoshita^1^, Tomoko Narita^1^, Ayako Masaki^3^, Asahi Ito^1^, Masaki Ri^1^, Shigeru Kusumoto^1^, Hirokazu Komatsu^1^, Hiroshi Inagaki^3^ and Shinsuke Iida^1^**

# **Extraction of genomic DNA**

Genomic DNA (gDNA) was extracted from formalin-fixed paraffin-embedded (FFPE) samples of lymph nodes for diffuse large B-cell lymphoma (DLBCL) diagnosis and bone marrow clot for acute promyelocytic-like leukaemia (APLL) diagnosis, using the GeneRead FFPE DNA Kit (QIAGEN, Hilden, Germany) according to the manufacturer’s instructions. gDNA was also extracted from frozen peripheral blood mononuclear cells for the diagnosis of CMMoL and buccal cells using the QIAamp DNA Mini Kit (QIAGEN) according to the manufacturer’s instructions.

## **Whole-genome sequencing**

Library preparation and sequencing were performed by BGI Genomics Co., Ltd. For FFPE-derived DNA samples, the library was prepared using the KAPA Hyper Prep Kit according to the BGI Genomics Co., Ltd.’s low-input whole-genome sequencing (WGS) library preparation protocol. For other DNA samples, the library was prepared according to the BGI Genomics Co., Ltd.’s standard WGS library preparation protocol. Sequencing of 100-bp paired-end reads was performed using the DNBSeq platform.

To detect somatic variants and structural variants (SVs), WGS reads were mapped to the reference genome of the GRCh37.p13/hg19 no-alt analysis set using speedseq align version 0.1.2 [1]. Somatic variants (single nucleotide variants and short indels) were detected using GATK Mutect2 version 4.1.9.0 [2] in tumour-normal mode with the following options: germline-resource, panel-of-normal, and --f1r2-tar-gz. A buccal swab sample was used as the matched normal sample. The variants were filtered using GATK FilterMutectCalls version 4.1.9.0, with the following options: contamination-table and ob-priors. The variants were annotated using SnpEff version 5.1d [3]. The following filtering criteria were applied to retain potentially significant variants: 1) the variant allele fraction was ≥0.30; 2) the depth for the variant allele was ≥8; and 3) the putative impact of the variant was HIGH or MODERATE (HIGH impact variant is assumed to have a high [disruptive] impact on the protein, probably causing protein truncation, low function, or triggering nonsense-mediated decay. The MODERATE impact variant is a non-disruptive variant that may alter protein effectiveness). The variants at the same position as the significant variants were rescued from the removed variants and included in the final set of variants (Table S1).

Somatic SVs were called using Manta version 1.6.0 [4] in the tumour-normal mode. The SVs were annotated using SnpEff version 5.1d [3]. The following criteria were applied to maintain potentially significant SVs: 1) the number of split reads supporting the SV was ≥6; 2) the number of paired-end reads supporting the SV was ≥4; 3) either or both SV breakpoints overlapped gene bodies; and 4) the SnpEff putative impact of the SV was HIGH. SVs whose breakpoint positions were the same as those of the significant SVs were rescued from the removed SVs and included in the final set of SVs (Table S2).

To detect germline variants, WGS reads from the buccal swab sample were mapped to the GRCh37.p13/hg19 no-alt analysis set genome using the speedseq align version 0.1.2 [1]. The base quality of the mapped reads was corrected using GATK ApplyBQSR version 4.2.5.0 [2]. Germline variants were detected using GATK HaplotypeCaller version 4.2.5.0. The germline variants were scored using GATK CNNScoreVariants version 4.2.5.0 with the option ‘--tensor-type read_tensor’ and filtered using FilterVariantTranches version 4.2.5.0 with the options ‘--info-key CNN_2D --invalidate-previous-filters true’. The variant sites in dbSNP (b155) were used as the known sites of common variation.

**Supplemental References**

1. Chiang, C., Layer, R.M., Faust, G.G., Lindberg, M.R., Rose, D.B., Garrison, E.P., Marth, G.T., Quinlan, A.R. & Hall, I.M. (2015) SpeedSeq: ultra-fast personal genome analysis and interpretation. Nat Methods, 12, 966-968.
2. van der Auwera, G. & O'Connor, B.D. (2020) Genomics in the Cloud: Using Docker, GATK, and WDL in Terra. O'Reilly Media, Incorporated.
3. Cingolani, P., Platts, A., Wang le, L., Coon, M., Nguyen, T., Wang, L., Land, S.J., Lu, X. & Ruden, D.M. (2012) A program for annotating and predicting the effects of single nucleotide polymorphisms, SnpEff: SNPs in the genome of Drosophila melanogaster strain w1118; iso-2; iso-3. Fly (Austin), 6, 80-92.
4. Chen, X., Schulz-Trieglaff, O., Shaw, R., Barnes, B., Schlesinger, F., Kallberg, M., Cox, A.J., Kruglyak, S. & Saunders, C.T. (2016) Manta: rapid detection of structural variants and indels for germline and cancer sequencing applications. Bioinformatics, 32, 1220-1222.
